# AmotL2 integrates polarity and junctional cues to modulate cell shape

**DOI:** 10.1038/s41598-017-07968-1

**Published:** 2017-08-08

**Authors:** Sara Hultin, Aravindh Subramani, Sebastian Hildebrand, Yujuan Zheng, Arindam Majumdar, Lars Holmgren

**Affiliations:** 1 Department of Oncology and Pathology, Cancer Centrum Karolinska (CCK), Karolinska Institutet, Solna, 171 76 Stockholm, Sweden; 2 Eli Lilly and Company, Lilly Corporate Center, Indianapolis, IN 46285 USA; 3 Department of Clinical Sciences, Intervention and Technology (CLINTEC), Karolinska Institutet and Division of Obstetrics and Gynecology, Karolinska University Hospital, Huddinge, Sweden

## Abstract

The assembly of individual epithelial or endothelial cells into a tight cellular sheet requires stringent control of cell packing and organization. These processes are dependent on the establishment and further integration of cellular junctions, the cytoskeleton and the formation of apical-basal polarity. However, little is known how these subcellular events are coordinated. The (Angiomotin) Amot protein family consists of scaffold proteins that interact with junctional cadherins, polarity proteins and the cytoskeleton. In this report, we have studied how these protein complexes integrate to control cellular shapes consistent with organ function. Using gene-inactivating studies in zebrafish and cell culture systems *in vitro*, we show that Par3 to be essential for localization of AmotL2 to cellular junctions to associate with VE/E-cadherin and subsequently the organization of radial actin filaments. Our data provide mechanistic insight in how critical processes such as aortic lumen expansion as well as epithelial packing into hexagonal shapes are controlled.

## Introduction

Epithelial cells bring support and structure to all the organs of the body, as well as functioning as efficient chemical and mechanical barriers. Further, they assure proper secretion, absorption and transport of water, nutrients and hormones. In a similar way endothelial cells line the blood vessels where they control diffusion of oxygen as well as protein transport^1, 2^. To form an epithelial sheet, individual cells typically pack into a tight layer of mainly hexagonally shaped cells. Both cell shape and tissue topology is dependent up on the length and stability of the cellular junctions^3^. All cell-cell junctions are composed of transmembrane proteins, eg. cadherins in adherens junctions and occludin/claudins/JAMs/Nectins in tight junctions. Intracellularly, the transmembrane proteins form complexes with adaptor proteins, which serve as signaling platforms and connect to the cytoskeleton^4^. Early formation of intercellular cadherin connections are supported by recruitment of actin-associated proteins and in return the cell-cell interactions stimulate actin filament polymerization^5, 6^.

Junction formation is essential for establishment of apical-basal polarity, by stimulating recruitment of the apical polarity complexes, Crb3/Pals1/PATJ and Par3/Par6/aPKC^7^. Correct localization of these protein complexes is crucial for proper development and function

of the apical cell domain^8^. Furthermore, Par3 has been shown to be essential for formation/ dynamics of tight and adherens junctions, and has also been connected to the actin cytoskeleton, by controlling activity of the small GTPase Rac1^9–11^.

We have previously shown that Par3 binds directly to the PDZ binding domain of the junction associated scaffold protein Angiomotin-Like-2 (AmotL2)^12^. AmotL2 belongs to the Angiomotin family, characterized by a N-terminal glutamine-rich domains of coil-coil repeats and a C-terminal PDZ-binding motif. The latter mediates binding to the PDZ domains of Par3, as shown by yeast two-hybrid analysis^12, 13^. We have recently shown that AmotL2 associates to the VE and E-cadherin complex to orchestrate the formation of radial actin fibers^14, 15^. Inactivation of AmotL2 in zebrafish and mouse endothelial cells results in loss of aortic lumen expansion, due to defects in actin cytoskeleton organization and further cell morphogenesis. Additonally, in epithelial cells, AmotL2 depletion affects cellular packing and morphogenesis^14^.

The establishment of different type of junctions and their components from which they consist were studied extensively. On the contrary, how the junction proteins come to localize to the correct membrane domain, and further how signals from junctional and polarity components intersect and transduce to finally merge into intra as well as supracellular signaling, is not yet clear.

We provide evidence for AmotL2 and Par3 being part of the same pathway regulating the actin cytoskeleton in both endothelia during aortic lumen formation and in skin epithelium. By doing so, we show the importance of linkage and coordination of polarity and cell-cell adhesion proteins for proper organization of trans-cellular actin filaments. Further, precise assembly of adherens junction associated actin filaments is required for hexagonal packing essential for the development and maintenance of a stable cellular sheet.

## Results

### Par3 is required for dorsal aorta formation in zebrafish

**Figure 1:**
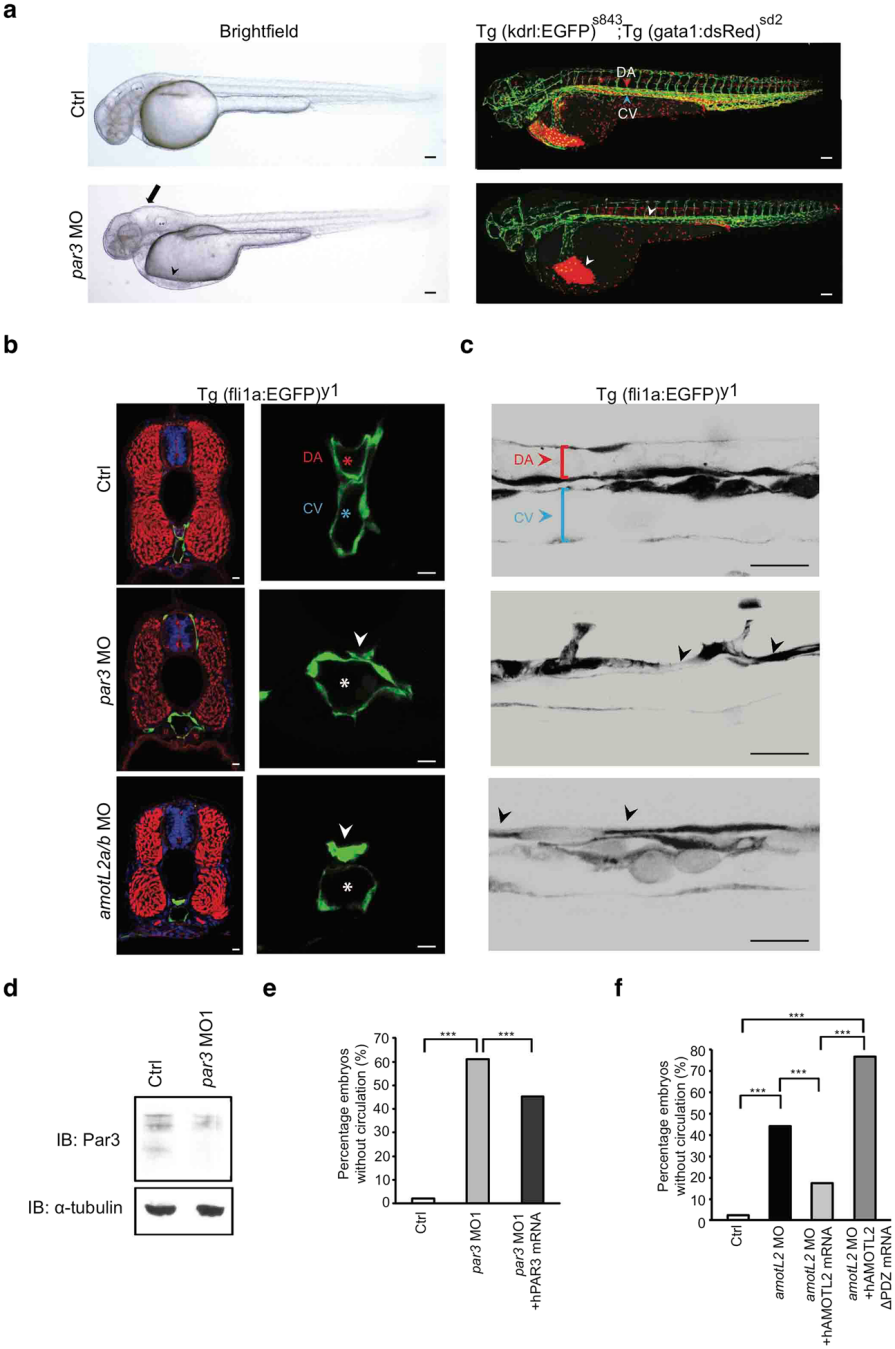
Par3 is required for aortic lumen formation during zebrafish development. (**a**) Brightfield (left) and fluorescence (right) images of double transgenic Tg (kdrl:EGFP)^s843^;Tg (gata1:dsRed)^sd2^, control (top) and *par3* MO (bottom) injected embryos at 48hpf. The *par3* morphants exhibited pericardial (arrowhead) and brain (arrow) edema. In addition, they lacked circulation, and no erythrocytes could be observed in the trunk vasculature (arrowhead). Scale bar, 100 μm. **(b)** Transverse sections of 48hpf Tg(*fli1a*:EGFP)^y1^ control (top row) and *par3* (mid row) morphants. Sections were stained with phalloidin for F-actin (red) and TO-PRO-3 iodide to visualize nuclei (blue). In control embryos patent lumens could be observed in both the dorsal aorta (red asterisk) and cardinal vein (blue asterisk). The *par3* morphants showed narrow aortic lumens with present constrictions (arrowhead), while the vein was lumenized (white asterisk). As a comparison sections of *amotL2a/b* embryos were also included (bottom row). Note the similarity of the aortic phenotype. Scale bar, 10 μm. (**c)** Sagittal view of the DA (red bracket/ arrowhead) and PCV (blue bracket/ arrowhead) in control, *par3* and *amotL2* morphants. Both *amotL2* and *par3* morphants show DA constrictions (arrowheads) and reduced DA diameter. Scale bar, 50 μm. (**d)** Western blot analysis showing the knock-down efficiency of the *par3* MO1. Alpha-tubulin was used to control for equal loading. **(e)** Quantification of the circulation defect in the *par3* morphants. The phenotype could be partially rescued by co-injecting the morpholino with a human *PAR3* mRNA. N(ctrl) = 147 embryos, N(*par3* MO) = 154 embryos, N(*par3* MO + *hPAR3* mRNA) = 117 embryos. *** p ≤ 0.001. (**f)** Rescue experiment of the circulatory phenotype observed in the *amotL2a/b* MO zebrafish embryos. The circulation could be restored by co-injecting the morpholinos with a human AMOTL2 mRNA, but not with an AMOTL2 mRNA lacking the c-terminal PDZ-binding motif. N(ctrl) = 100 embryos, N(AmotL2 MO) = 111 embryos, N(AmotL2 MO + hAMOTL2 mRNA) = 141 embryos, N(AmotL2 MO + hAMOTL2 ΔPDZ mRNA) = 66 embryos, *** p ≤ 0.001.

Similar to AmotL2, Par3 has been reported to be important for aortic lumen formation, as expression of Par3 was shown to rescue aortic defects caused by β1-integrin deletion in mice^15, 16^. A zebrafish orthologue to *Par3* has been reported, which present high homology to mammalian Par3 regarding amino acid identity and organization of functional domains^17^. To analyze the role of Par3 during vascular development in zebrafish, we used anti-sense morpholino oligos to functionally inactivate Par3^17^. As higher doses of *par3* morpholinos were previously shown to cause severe brain and eye defects, the concentrations were reduced to allow detection of potential vascular defects^17^. At 48hpf the *par3* morphants presented with pericardial and brain edema and further lacked circulation, the latter similar to what was observed in the *amotL2a/b* MO (Fig. 1a). Accordingly, using the double transgenic *Tg(gata1:dsRed)* ^*sd2*^ *); Tg(kdrl:EGFP)* ^*s843*^ embryos, we could detect pericardial accumulation of the dsRed positive erythrocytes (Fig. 1a). In contrast, the control embryos had established circulation and dsRed positive erythrocytes were found circulating within the EGFP expressing vasculature (Fig. 1a). To study the circulatory phenotype more closely, 48hpf *Tg(fli1a:EGFP)* ^*y1*^ control and *par3* morphants embryos were analyzed by vibratome sectioning. In accordance with previously published findings in mice, a lumen defect was observed in the dorsal aorta, while the vein was lumenized (Fig. 1b,)^16^. The dorsal aorta was narrow, with prominent constrictions (Fig. 1c), and interestingly the phenotype phenocopied our previously observed findings in the *amotL2* MO zebrafish embryos (Fig. 1b,c,)^15^. The efficiency of the *par3* morpholinos to reduce Par3 protein levels was confirmed by western blot (Fig. 1d and Supplementary Fig. 3a). The same circulatory phenotype could be obtained using two different *par3* morpholinos and the phenotype could further be rescued by co-injecting the morpholinos with a human *PAR3* mRNA. This argues for specificity of the detected phenotype (Fig. 1e, Supplementary Fig. 3a–c). The association between the two proteins was further strengthened by the observation that rescue of the *AmotL2* MO circulatory phenotype was dependent on the PDZ-binding domain, previously shown to interact with Par3 (Fig. 1f,)^12^.

### Par3 controls actin filament organization during development of zebrafish skin

**Figure 2:**
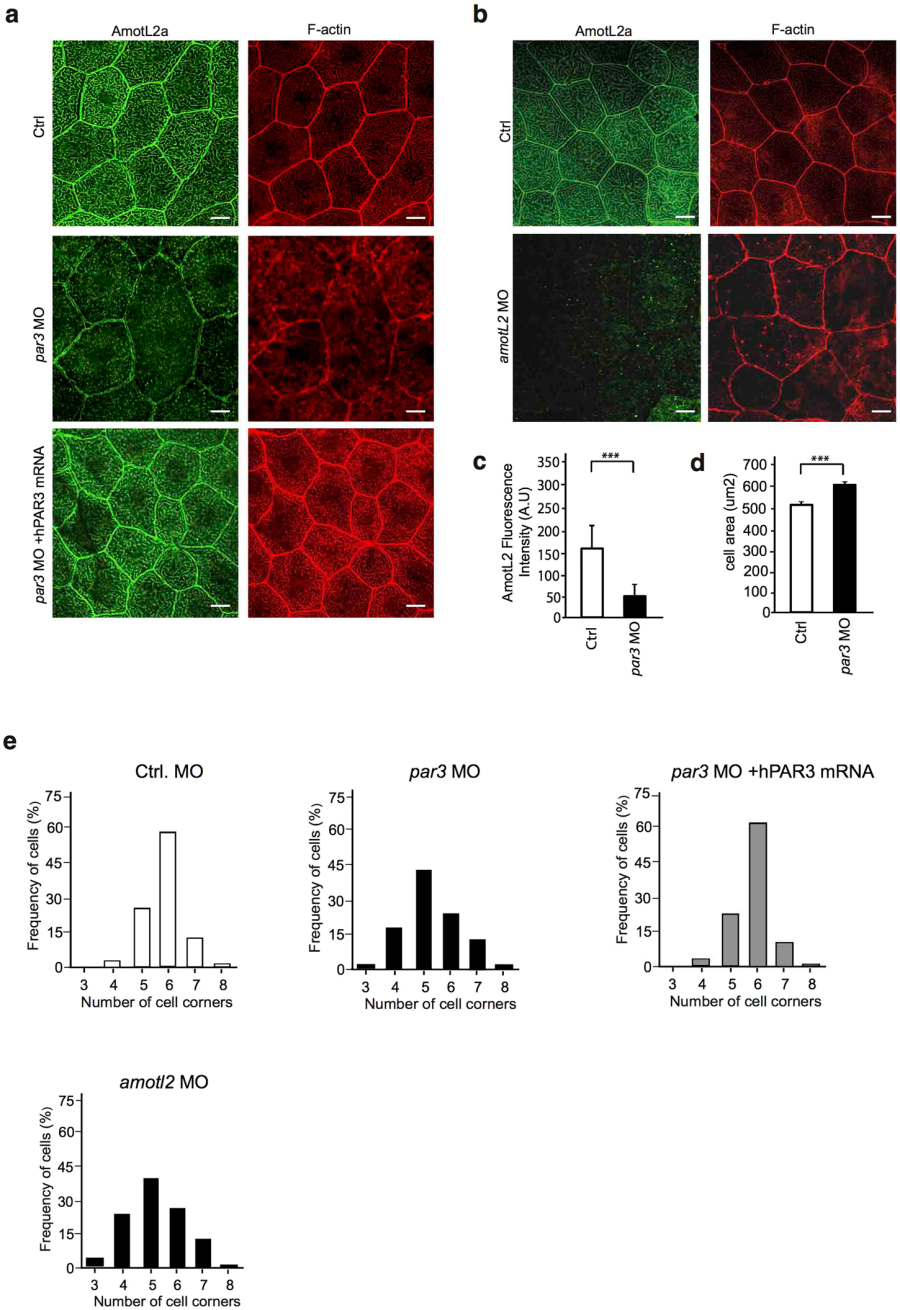
Par3 controls actin filament architecture and morphology in epithelial cells of zebrafish skin. (**a**) Immunofluorescence staining of AmotL2a (green) and F-actin (red) in skin of control (first panel) and *par3* MO (second panel) treated zebrafish embryos at 34hpf. In control embryos, AmotL2a localizes to cell-cell junctions, as well as actin positive apical microridges. In the absence of Par3 those actin structures are lost and in addition AmotL2a expression at cell-cell junctions is reduced. The actin filament phenotype could be rescued by co-injecting the morpholino with a human *PAR3* mRNA (third panel). (**b**) As a comparison pictures of skin from *amotL2a/b* morphants AmotL2a (green) and F-actin (red) show similar to what was observed in the *par3* morphants, the embryos deficient of AmotL2 showed loss of cytoplasmic actin filaments. Scale bar 10 um. (**c)** Quantification of fluorescence intensity of AmotL2 at cell junctions in control and *par3* MO injected embryos. N (ctrl) = 100 cells, N (*par3* MO) = 100 cells. P values ctrl vs *par3* MO < 0.0001 as calculated by unpaired t test. (**d**) Quantification of cell area in control and *par3* MO injected embryos. N (ctrl) = 100 cells, N (*par3* MO) = 100 cells. P values ctrl vs *par3* MO < 0.0001 as calculated by unpaired t test. **(e)** Quantification of cell geometry in control, *par3* morphants and *amotl2* as well as embryos co-injected with *par3* MO and human *PAR3* mRNA. N (ctrl) = 200 cells, N (*par3* MO) = 200, N(*amotl2 MO) = *200 cells cells, N(par3 MO + hPAR3 mRNA) = 200 cells. p value ctrl vs. par3 MO < 0.0001. p-value par3 MO vs. par3 MO + hPAR3 mRNA < 0.00001, ctrl vs par3 MO + hPAR3 mRNA = 1. Each experiment was repeated at least three times.

In endothelial cells, AmotL2 is required for connection of radial actin filaments to VE-cadherin^15^. Amotl2a is also expressed in the zebrafish skin cells of the developing epidermis, where it is localized to cell-cell junctions, as well as actin filaments (Fig. 2a). We have recently shown that, in analogy with endothelial cells, AmotL2 associates to E-cadherin in zebrafish, mouse and human cells and is required for epithelial geometry and blastocyst hatching^14^. In zebrafish skin, depletion of AmotL2 resulted in loss of cytoplasmic filaments and change in cell area and epithelial packing as previously published by Hildebrand *et al*. (Fig. 2b)^14^. We went on to investigate whether depletion of Par3 in the zebrafish skin would affect AmotL2 junctional organization and actin filament structures. The phenotype of *par3* MO did partially overlap with that of *amotl2* MO in that cytoplasmic actin filaments were lost and cell area was significantly increased (Fig. 2a and d). Furthermore a similar defect in geometrical cell packing was observed in the *par3* and amotl2 morphants (Fig. 2e). Analysis of AmotL2a localization showed a significant decrease of AmotL2 staining in cellular junctions (Fig. 2a,c). In comparison to *amotl2* morphants, the *par*3 MO phenotype was more severe as junctions appeared less intact (Fig. 2a). Both the increased cell area and the altered polygonal distribution could be rescued by co-injection with a human *PAR3* mRNA (Fig. 2a,e). In accordance with the results from the Par3 depletion in vasculature, the *par3* MO skin phenotype strongly mimics the phenotype observed in the *amotL2* morphants (Fig. 2,)^14^.

### AmotL2 and Par3 form a protein complex controlling actin filament induction from adherens junctions

**Figure 3:**
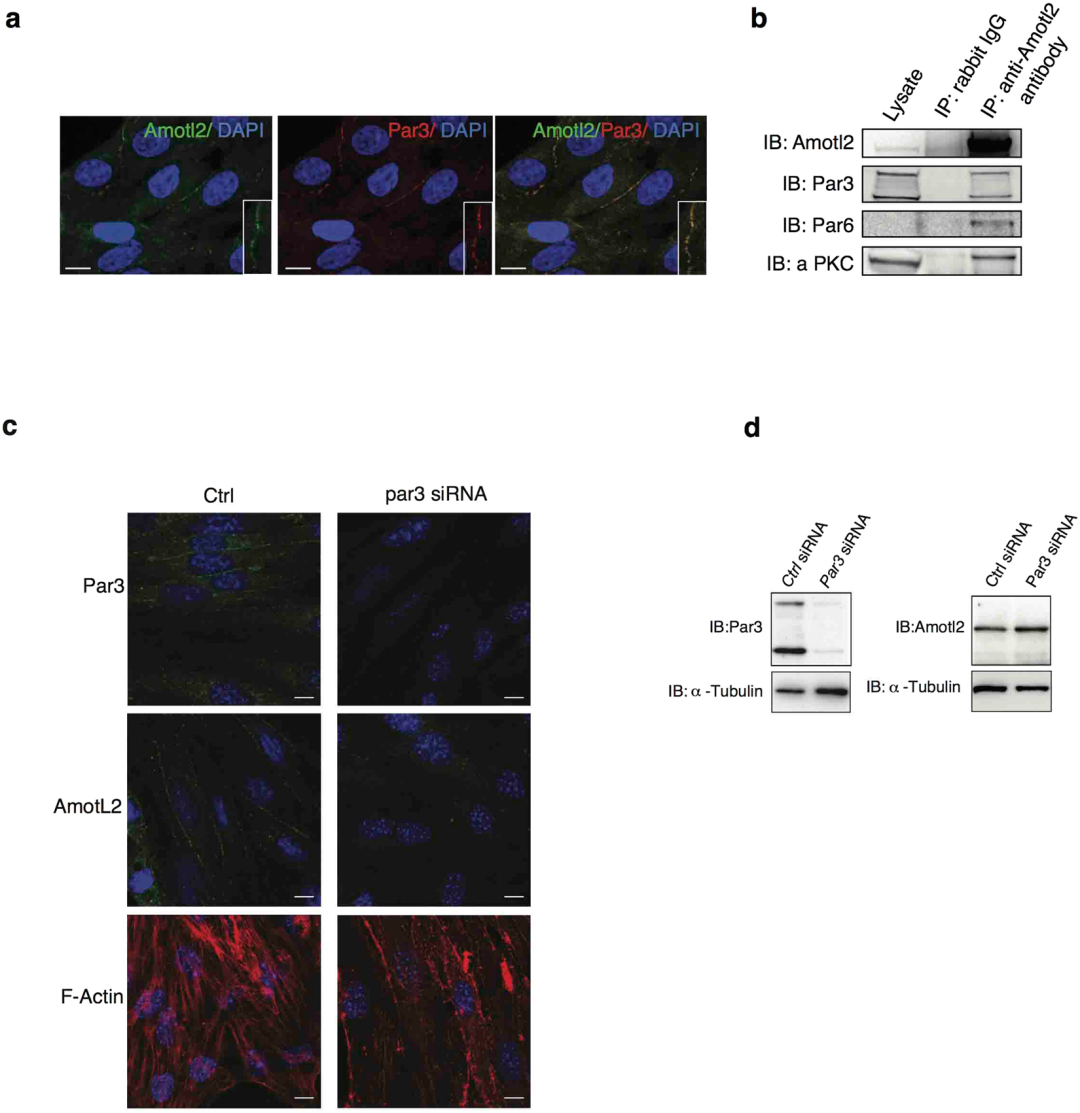
AmotL2 and Par3 associate to a common protein complex at cell-cell junctions. (**a**) Immunofluorescence staining showing co-localization of AmotL2 (green) and Par3 (red) at cell-cell junctions in bovine capillary endothelial (BCE) cells. Inset shows high magnification of cell-cell junction. Scale bar, 10 um. (**b**) Co-immunoprecipitation analysis reveal association of AmotL2 to the Par3/Par6/aPKC polarity complex in MileSven1 pancreatic islet endothelial (MS-1) cells. Both the 100 kDa and 150 kDa isoforms of Par3 were found to interact with AmotL2. (**c**) Immunofluorescence stainings of MS-1 cells, treated with control (left panel) or *Par3* (right panel) siRNA, respectively. Both Par3 and AmotL2 (green) are localized to the cell-cell junctions in the control cells. In absence of Par3, AmotL2 junctional staining is lost, and actin filaments (red) are disrupted. Nuclei were visualized with TO-PRO-3 iodide. Scale bar, 10 μm. (**d**) Western blot analysis of siRNA depletion of Par3 in MS-1 cells. Par3 depletion did not affect steady-state levels of AmotL2.

The expression pattern of AmotL2 and Par3 was compared using immunofluorescence staining. As expected, expression of both proteins could be detected at the cell-cell junctions in Bovine Capillary Endothelial (BCE) cells (Fig. 3a). Co-immunoprecipitation analysis showed AmotL2 and Par3 to be part of the same protein complex (Fig. 3b). In addition, the role of Par3 in controlling actin cytoskeleton architecture could be confirmed by small interfering RNA (siRNA) analysis in Mile Sven-1 mouse pancreatic islet endothelial cells (MS-1) *in-vitro*. Here, depletion of Par3 resulted in loss of radial actin filaments but did not affect steady-state levels of AmotL2 (Fig. 3c,d).

### Par3 is essential for junctional localization of AmotL2

**Figure 4:**
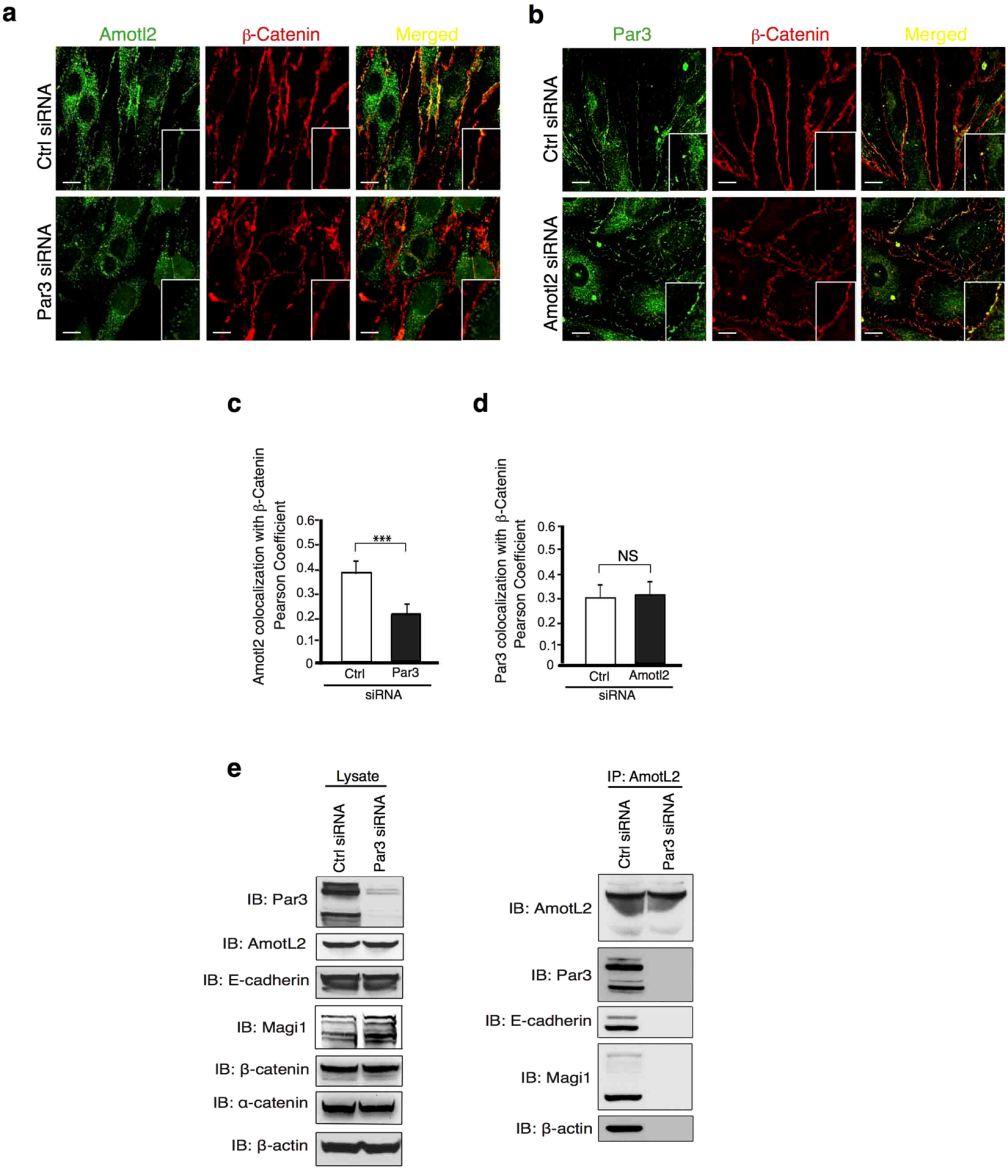
AmotL2 localization to cell-cell junctions is Par3 dependent. (**a,b**) In MS-1 cells treated with control siRNA, both AmotL2 and Par3 (green) localize to β-catenin (red) positive cellular junctions. In *Par3* siRNA treated cells, AmotL2 protein was absent from β-catenin positive junctions. In MS-1 cells treated with *AmotL2* siRNA, Par3 junctional localization was unaffected. Insets show high magnification of cell-cell junctions. Scale bar 10 μm. (**c**) Quantification of AmotL2 co-localization to β-catenin in control and *Par3* siRNA treated MS-1 cells. N(control siRNA) = 64 cells, N(*Par3* siRNA) = 42 cells, p = 8.80 × 10^−5^. (**d**) Quantification of Par3 co-localization to β-catenin in control and *AmotL2* siRNA treated MS-1 cells. N(control siRNA) = 38 cells, N(*AmotL2* siRNA) = 70 cells, p = 0.87, Error bars indicate standard deviation (s.d.), N.S. = Non Significant, ***p ≤ 0.001. (**e**) co-IP demonstrating the association of both Par3 and AmotL2 to the E-cadherin protein complex. Interaction of AmotL2 to E-cadherin and MAGI-1/ β-actin was dependent on Par3.

To further analyze the functions of the AmotL2-Par3 interaction, we used siRNA to target the two proteins individually in MS-1 cells. Interestingly, the junctional localization of AmotL2 was lost in Par3 siRNA-depleted cells as AmotL2 localization was not overlapping with β-catenin at cell-cell junctions (Fig. 4a,c). In contrast, silencing AmotL2 did not affect spatial distribution of Par3, which was localized to cell-cell junctions both in control and *AmotL2* siRNA treated cells (Fig. 4b,d). No effect on total protein levels of par3 was observed in MS-1 cells, arguing for an effect mainly on the localization (Supplementary Fig. 1a). These findings suggest that Par3 plays an important role in targeting AmotL2 to the cellular junction, whereas AmotL2 is dispensable for Par3 localization. The same result could be obtained using HaCaT (human keratinocytes) cells, implying that this is a conserved mechanism among tissues (Supplementary Fig. 1b). Further, the results were strengthened by analysis of Par3 localization in zebrafish skin. By injecting mRNA encoding human Par3-GFP^18^, alone or in combination with *AmotL2* morpholinos, we were able to study the effect of AmotL2 on Par3 localization. Par3-GFP could be detected in zebrafish skin cells where it was localized to cell-cell junctions independent of AmotL2 levels (Supplementary Fig. 2a). We conclude that Par3 is required for AmotL2 junctional localization and the formation of radial actin filaments.

### Par3 is essential for AmotL2/E-cadherin interaction

**Figure 5:**
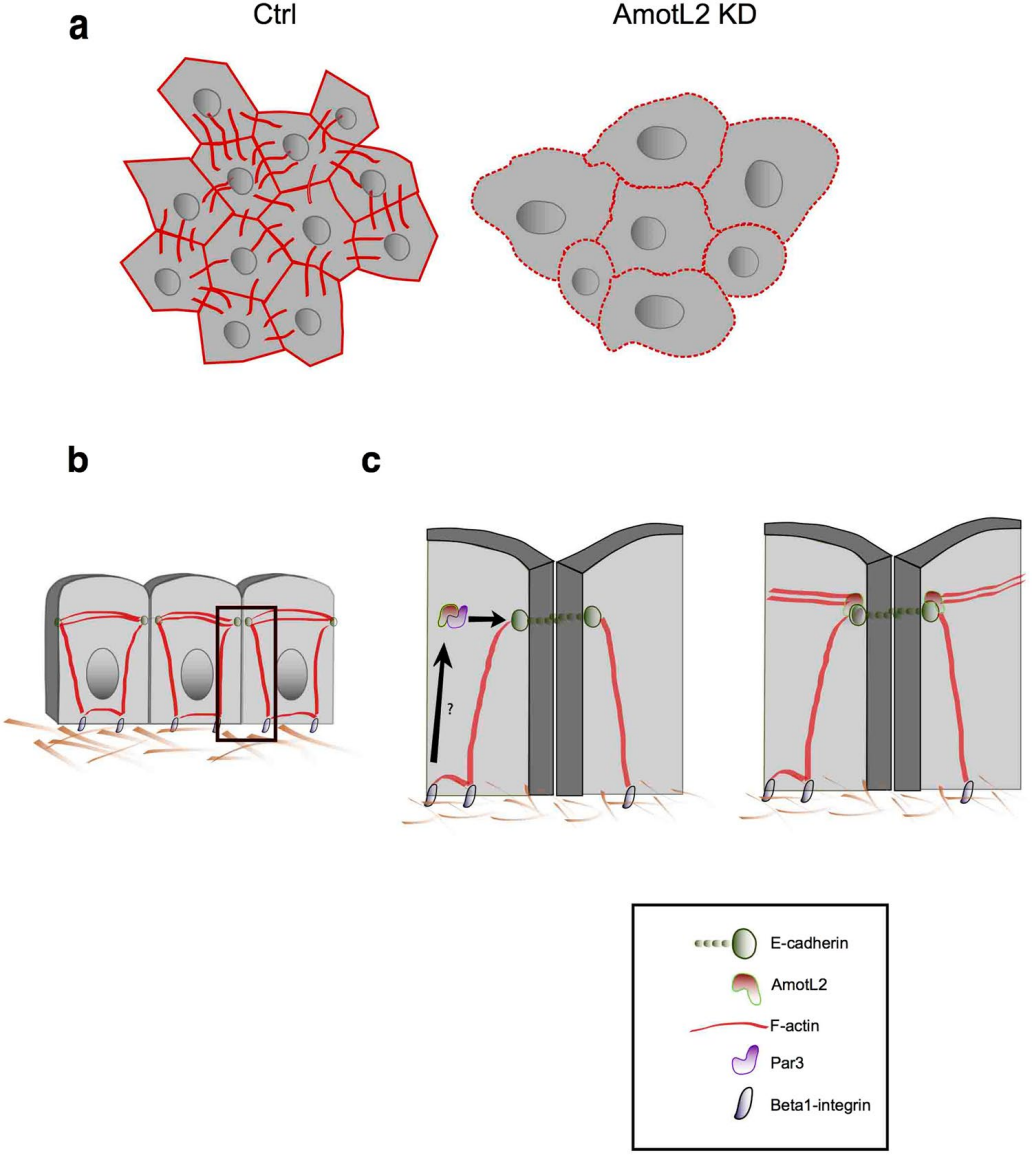
Hypothetical model of the Par3/AmotL2/(V)E-cadherin interaction to mediate actin filaments during cell morphogenesis and sheet formation. (**a**) Cells of the epidermis of control zebrafish embryos form a tight sheet consisting of mainly hexagonal and pentagonal cells, with structured actin cytoskeleton architecture. Cells of the epidermis of Par3 /AmotL2 KD embryos show altered morphology and increased area. The actin cytoskeleton is visibly disorganized. (**b**) Epithelial cells form E-cadherin based adherens junction, which associate to radial actin filaments, as well as cortical actin and actin filaments connected to the focal adhesions. (**c**) Hypothetically, AmotL2 is targeted to the apical adherens junctions by the polarity protein Par3. At the junctions AmotL2 associates with the (V) E-cadherin protein complex to mediate formation of radial actin filaments, and transmission of force in between cells.

We have previously shown that AmotL2 was important for VE/E-cadherin dependent actomyosin contractility^14, 15^. We continued to analyze the role of Par3 in the interaction of AmotL2 with the E-cadherin adherens junction protein complex. Interestingly, silencing of Par3 using siRNAs in HaCaT cells totally abrogated the association of AmotL2 to E-cadherin, MAGI-1 and β-actin (Fig. 4e) as evaluated by co-IP. In conclusion, these findings suggest the AmotL2/ E-cadherin interaction to be mediated at the cell membrane. The components of the protein complex would be individually targeted to the same membrane domain, where they later associate to allow induction of actin filaments and connection of neighbouring cells (Fig. 5).

## Discussion

Despite extensive studies regarding the composition and organization of adhesive contacts, it has not yet been revealed how the junctional proteins are targeted to the correct membrane domain. In this report, present data connecting Par3 to the junctional localization of AmotL2 and subsequent organization of actin filaments. By bridging adherens junctions and its associated actin filaments to polarity signaling, we propose Par3 and AmotL2 to form a molecular complex, translating polarity cues into co-ordinated actomyosin organization.

Recently, both Par3 and AmotL2 have been shown to interact with VE/E-cadherin at the adherens junction^10, 14, 15^. In this study, we demonstrate Par3 and AmotL2 to be part of a common adherens junction associated signaling complex. Silencing of Par3 was shown to cause disorganization of radial actin cytoskeleton as previously observed in AmotL2 knockdown studies. In more detail, the cells were lacking transcellular actin filaments, while the cortical actin appeared intact. As a consequence, the cells present with an altered morphology and the hexagonal patterning was lost. However, since VE/E-cadherin is associated to both transcellular actin filaments and cortical membrane associated actin, we cannot exclude loss of function of the latter as a contributing factor to the altered cell shape observed in AmotL2 and Par3 KD^3^.

Interestingly, when silencing Par3, we observed an apparent re-localization of AmotL2 from cell-cell junctions to cytoplasmic vesicles. On the contrary, we were not able to observe any difference in total AmotL2 protein levels, arguing against a control mechanism at the transcriptional level. Silencing of Par3 did not seemingly affect junctional localization of VE/E-cadherin or the associated catenins. Instead we report, Par3 is essential for proper localization of AmotL2 to the correct membrane domain. This is in line with previous findings presenting a difference in E-cadherin stability, but not localization, in the absence of Par3^11^.

Traditionally Par3 has been associated to Par6 and aPKC, playing a key role during epithelial/endothelial cell apical-basal polarization. As we have been unable to show any effect of AmotL2-silencing on apical-basal polarity, we propose Par3 to be part of two functionally distinct protein complexes. Accordingly, we here describe the association of Par3 to AmotL2 and VE-cadherin, forming a novel adherens junction associated protein complex controlling the actin cytoskeleton. This is in agreement with findings by Iden *et al*. p^10^, providing evidence for a distinct subset of VE-cadherin interacting with PAR proteins in endothelial cells.

As Par3 has been suggested to be associated to the Extracellular Matrix (ECM) through an interaction with β1-integrin^16^, it is tempting to speculate, that this interaction would further localize AmotL2 to the adherens junctions. Potentially, mechanosensing through β1-integrins at the focal adhesions could, through Par3 signaling, induce re-localization of AmotL2 from cytoplasmic vesicles to the adherens junctions. Once in its correct location, the AmotL2/cadherin/catenin protein complex would allow induction of actin filament formation from the adherens junction, and further reinforcement of the junction to better withstand external force.

Taken together, we have revealed a role for the polarity protein Par3 in targeting AmotL2 to cell-cell junctions. Once at the site, AmotL2 and Par3 interact with VE/E-cadherin and associated catenins, to induce transcellular actin filaments anchoring neighbouring cells, and allowing proper cell sheet formation. We hypothesize the AmotL2-Par3 junctional recruitment to be activated through β1-integrin signaling at the focal adhesions. Converting polarity signals into actin cytoskeletal modifications, we propose this signaling pathway to be part of a machinery enabling sensing of the micro-environment.

Since the actin fibers are not limited to one cell length, but instead span through several cells, the force initially sensed at the basal cell-ECM interface can be transmitted in between cells, coordinating the neighboring cells into a synchronized tissue. Hence, mislocalization of junctional components might have big implication both on cell and tissue level. Accordingly, strengthening of junctions and connection in between cells is commonly lost during tumor progression. Hence, this study can be of importance not only for understanding of developmental processes but also for advances within tumor biology.

## Materials and Methods

### Zebrafish strains

Zebrafish were raised and housed under standard conditions and in agreement with the regulations set out by the Swedish Board of Agriculture for the use of laboratory animals in scientific research. The following previously described transgenic lines were used in this study:

Tg(kdrl:EGFP)^s843^ ^19^

Tg(gata1:dsRed)^sd2^ ^20^

Tg(fli1a:EGFP)^y1^ ^21^

All experiments were performed in accordance with relevant guidelines and regulations. All experimental protocols were approved by the regional ethics board (Jordbruksverket.se).

### DNA, RNA and morpholino injections into zebrafish embryos

Morpholinos were purchased from Gene Tools (Philomath, Oregon, USA). The following morpholinos were used in this study:

amotl2a MO 5′-CTGATGATTCCTCTGCCGTTCTCAT-3′^14, 15^

amotl2b MO 5′-TGAGTATTTATGATCTGAGCTGAAC-3′^14, 15^

par3 MO1 5′-TCAAAGGCTCCCGTGCTCTGGTGTC-3′^17^,

par3 MO3 5′-TCCCGTGCTCTGGTGTCAAGATCAT-3′^17^

control 5′-CCTCTTACCTCAGTTACAATTTATA-3′

The amotl2a morpholino was injected at 1.5 ng per embryo and the amotl2b morpholino was injected at 3 ng per embryo. Morpholino-injected zebrafish embryos were maintained at 28 °C in standard E3 water supplemented with 0.003% phenyl-2-thiourea.

mRNA encoding wildtype or mutant human *AMOTL2* or *PAR3* were synthesized using the SP6 Message Machine kit (Ambion, Austin, TX, USA), and 50 pg per embryos were co-injected with morpholinos for rescue experiments. Embryos were fixed in 4% PFA at 28hpf for cell area analysis and at 34 hpf quantification of cell corners. For visualization of Par3-GFP^18^, living embryos were sedated with tricaine and mounted into 1% low-melt agarose on a coverslip. All experiments were performed in accordance with relevant guidelines and regulations. All experimental protocols were approved by the regional ethics board (Jordbruksverket.se).

### Immunofluorescence staining of zebrafish embryos

PFA fixed zebrafish embryos were washed in PBS-T, and further permeabilized in PBS-T containing 0.5% TritonX-100 for 30 minutes at room temperature (RT). Blocking was performed for two hours at RT, in a solution comprised of 0.1% TritonX-100, 5% Normal Goat Serum (NGS) and 1% BSA, all diluted in PBS-T. Antibodies were also diluted in this solution and antibody incubations were performed over night in +4 **°**C. After antibody incubations embryos were washed in PBS-T for 2 h (after primary antibody) and 4 h (after secondary antibody). For vibratome sections fixed embryos were mounted in 4% low-melt agarose and sectioned with a Leica Vibratome at a thickness of 200μm. Sections were stained with TexasRed-X Phalloidin (1:200, #T7471, Invitrogen) for visualizing F-actin and TO-PRO-3 iodide (1:1000, #T3605, Invitrogen) for labeling of the nuclei. Paralogue-specific epitopes were chosen for the production of anti-amotL2a antibodies. Affinity-purified rabbit polyclonal antibodies against the zebrafish amotL2a protein were made using the antibody production services of Innovagen (Lund, Sweden). For amotL2a, antibodies were made against the C-terminal epitope:

NH2-CQKAPSAVDLFKGVDDVSAE-COOH (1:200). All experiments were performed in accordance with relevant guidelines and regulations. All experimental protocols were approved by the regional ethics board (Jordbruksverket.se).

### Immunofluorescence staining of cells

Cells were washed in 1xPBS and fixed in 4% PFA for 10 minutes at room temperature (RT). After washing, permeablization was performed with TritonX-100 for 1 minute in RT. The cells were washed again and blocked for 1 h in 5% horse serum in PBS. Antibodies were diluted in the blocking solution, and all incubations were performed at RT. Incubations were 1–2 hours for primary antibodies and 45 minutes for secondary antibodies. The following antibodies and dyes were used:

anti-Par3 (1:100, rabbit polyclonal, #07–330, Millipore), anti-β-catenin (1:100, mouse monoclonal, #610154, BD Transduction Laboratories), TO-PRO-3 Iodide (1:1000, #T3605, Invitrogen), Phalloidin-TexasRed (1:200, #T7471, Invitrogen), AlexaFlour 488/568 secondary antibodies (1:1000, Invitrogen). AmotL2 was detected with a rabbit affinity-purified antibody against the C-terminal motif of human amotL2; NH2-CLDSVATSRVQDLSDMVEILI-COOH (1:50). Images were taken on a Zeiss LSM700 confocal microscope. Image J was used to measure the fluorescence intensities and to process the images acquired.

### Co-Localization Analysis

Co-localization of (AmotL2/Par3) with β**-**Catenin was calculated using the Coloc module of Imaris (Bitplane AG). The images (Z-Stacks) obtained from the confocal (Zeiss LSM700) microscope were loaded in Imaris and the automatic threshold function in the software was chosed for the green and red channels. Then the voxel intensities of the two channels green (AmotL2/Par3) and red (**β-**Catenin) in the junctions were used to calculate the Pearson Correlation Coefficient.

### siRNA transfections

Cells were seeded on glass slides (BD Falcon Cultureslides BD Biosciences) coated with 1% BD Matrigel Basement Membrane Matrix (BD Biosciences) in growth medium without antibiotics. Next day the growth medium was exchanged for OPTI-MEM I Reduced Serum Media (Invitrogen) and siRNA transfections were performed using Oligofectamine Transfection Reagent (Invitrogen) according to the manufacturer’s protocol. Four hours after transfection serum was added to a final concentration of 20%. Cells were allowed to grow for 72 h prior to the evaluation by immunofluorescence staining or western blot.

The following siRNA’s were used:

siGENOME siRNAs against mouse *AmotL2* (0.32 μM, M-062016–01, Dharmacon/Thermo Scientific).

siGENOME siRNAs against mouse *Par3* (0.64 μM, M-040036–01, Dharmacon/Thermo Scientific).

non-targeting siRNA Pool #2 (0.32/ 0.64 μM, D 001206–14, Dharmacon/Thermo Scientific).

siGENOME siRNAs against human *AMOTL2* (0.32 μM, M-013232–00, Dharmacon/Thermo Scientific).

ON-TARGETplus siRNAs against human *PAR3* (0.32 μM, L-015602–00, Dharmacon/Thermo Scientific).

### Immunoprecipitation and western blotting

For immunoprecipitation analysis cell lysates were prepared using a buffer containing 50 mM Tris-HCL pH 7.6, 150 mM NaCl, 1 mM EDTA, 1% Nonident P40 and 1 x protease inhibitor (Roche). Lysates were incubated with a rabbit affinity-purified antibody against the C terminal motif of human amotL2; NH2-CLDSVATSRVQDLSDMVEILI-COOH or with rabbit immunoglobulins as a control. Antibody incubations were performed for 2 hours under rotation at 4°C. Protein G Sepharose beads (GE Healthcare) were added for additional 2 hours. Beads were washed 4 times with lysis buffer and heated for 10 min at 95 °C in 2 x LDS sample buffer (Novex) containing 10% sample reducing agent (Novex). Immunocomplexes bound to the beads were separated by SDS-PAGE and AmotL2 binding proteins were detected by western blot. Fractions of whole cell lysates were western blotted for evaluation of IP protein input level.

The following antibodies were used: anti-Par3 (rabbit polyclonal. #07–330, millipore), anti-Par6 (rabbit polyclonal, #ab45394–100, abcam), anti-aPKC (anti-rabbit, #sc-216, santa cruz), anti-E-cadherin (1:500, mouse monoclonal, #610183, BD Transduction Laboratories), anti-β-catenin (1:500, #610154, 14/beta-catenin, BD Transduction Laboratories), anti-α-catenin (1:500, #610193, 5/alpha-catenin, BD Transduction Laboratories), anti-MAGI1 (1:500, **#**WH0009223M3, 7B4, Sigma), anti-β-actin (1:20000, #ab3280, ALTN05CC4, Abcam), Tubulin (Sigma, T5168). AmotL2 was detected with a rabbit affinity-purified antibody against the C-terminal motif of human amotL2; NH2-CLDSVATSRVQDLSDMVEILI-COOH (1:500).

### Statistical analysis

At least data from three independent experiments are presented as mean ± s.d. Statistical analysis. For zebrafish experiments, over 60 larvae were analysed per condition. This gave 80% power to detect a 20% difference with 95% confidence. For categorical qualitative analysis χ^2^ test was used and for quantitative data evaluation two-tailed student’s t-test was performed. Pattern distribution was analysed with Non-parametric unpaired t test.

## Electronic supplementary material

Supplementary Information
