# Supplementary material for: AmotL2 integrates polarity and junctional cues to modulate cell shape
Source: Sci Rep. 2017 Aug 8;7:7548. doi: 10.1038/s41598-017-07968-1 (PMC5548744; doi:10.1038/s41598-017-07968-1)
Supplement: Supplementary file 1 — Supplementary Information [file 41598_2017_7968_MOESM1_ESM.pdf]

Supplementary information file for the manuscript:

AmotL2 integrates polarity and junctional cues to modulate cell shape.

Sara Hultin[1#], Aravindh Subramani[1#], Sebastian Hildebrand[1,3], Yujuan Zheng[1], Arindam Majumdar[1, 2], Lars Holmgren[1*]

[1]Department of Oncology and Pathology, Cancer Centrum Karolinska (CCK), Karolinska Institutet, Solna 171 76, Stockholm, Sweden

[2] Eli Lilly and Company, Lilly Corporate Center, Indianapolis, IN 46285 U.S.A

[3]Department of Clinical Sciences, Intervention and Technology (CLINTEC), Karolinska Institutet and Division of Obstetrics and Gynecology, Karolinska University Hospital, Huddinge, Sweden

[*]Lars Holmgren, E-mail: lars.holmgren@ki.se

# These authors contributed equally to this work

Supplementary Figure 1. Par3 controls actin filament architecture in endothelial cells *in-vitro*. **(a)** Western blots showing efficient siRNA knock-down of AmotL2 in MS-1 cells. Knock-down of AmotL2 does not affect total levels of Par3. **(b)** Quantification of AmotL2 co-localization to β-catenin in control and *PAR3* siRNA treated HaCat cells. N(control siRNA)=50 cells, N(Par3 siRNA)=50 cells, Error bars indicate standard deviation (s.d), *** $P \leq 0.001$.

Supplementary Figure 2. Par3 is efficiently localized to cell-cell junctions independent of AmotL2. (a) Expression of Par3-GFP in control and *AmotL2a/b* MO treated zebrafish embryos. Par3 show expression at cell-cell junctions independent of AmotL2. Scale bar 10 mm.

Figure 3. The same phenotypes of the zebrafish vasculature and skin are observed using two different *par3* morpholinos. (**a)** Western blot showing the knock-down efficiency using *par3* MO3. α-tubulin was used to control for equal loading. (**b)** Quantification of the circulation defect in the *par3* MO3 embryos. The phenotype could be partially rescued by co-injecting the morpholino with a human *PAR3* mRNA. N(ctrl)= 100 embryos, N(*par3* MO)= 104 embryos, N(*par3* MO+ *hPAR3* mRNA)= 124 embryos. *** $P \leq 0.001$**. (c)** Immunofluorescence images showing AmotL2a and F-actin staining of the epidermis of ctrl (top panel), *par3* MO3 (mid panel) and *par3* MO3 + hPAR3 mRNA (lower panel) injected zebrafish embryos. The structure of the actin cytoskeleton is disrupted in the *par3* MO3 injected embryos, correlating with and altered cell shape. Both the cell morphology and the actin filaments could be restored co-injecting the morpholino with a human *PAR3* mRNA.

a

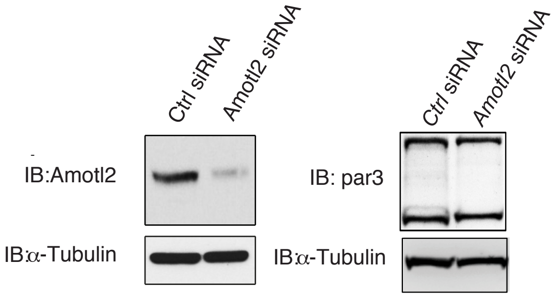

b

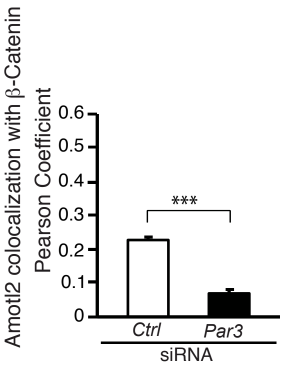

Hultin et al, Supplementary Figure 1.

a

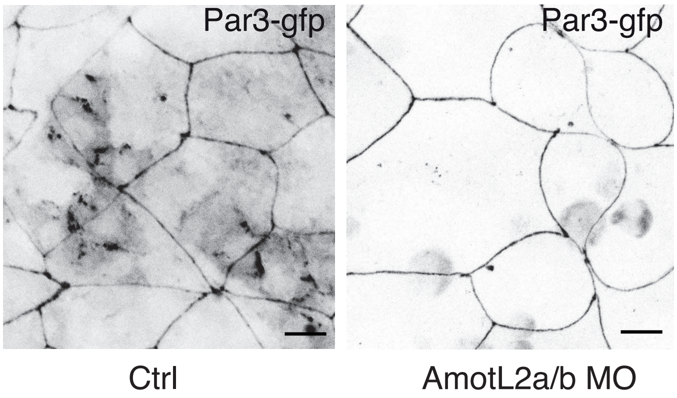

Hultin et al, Supplementary Figure 2

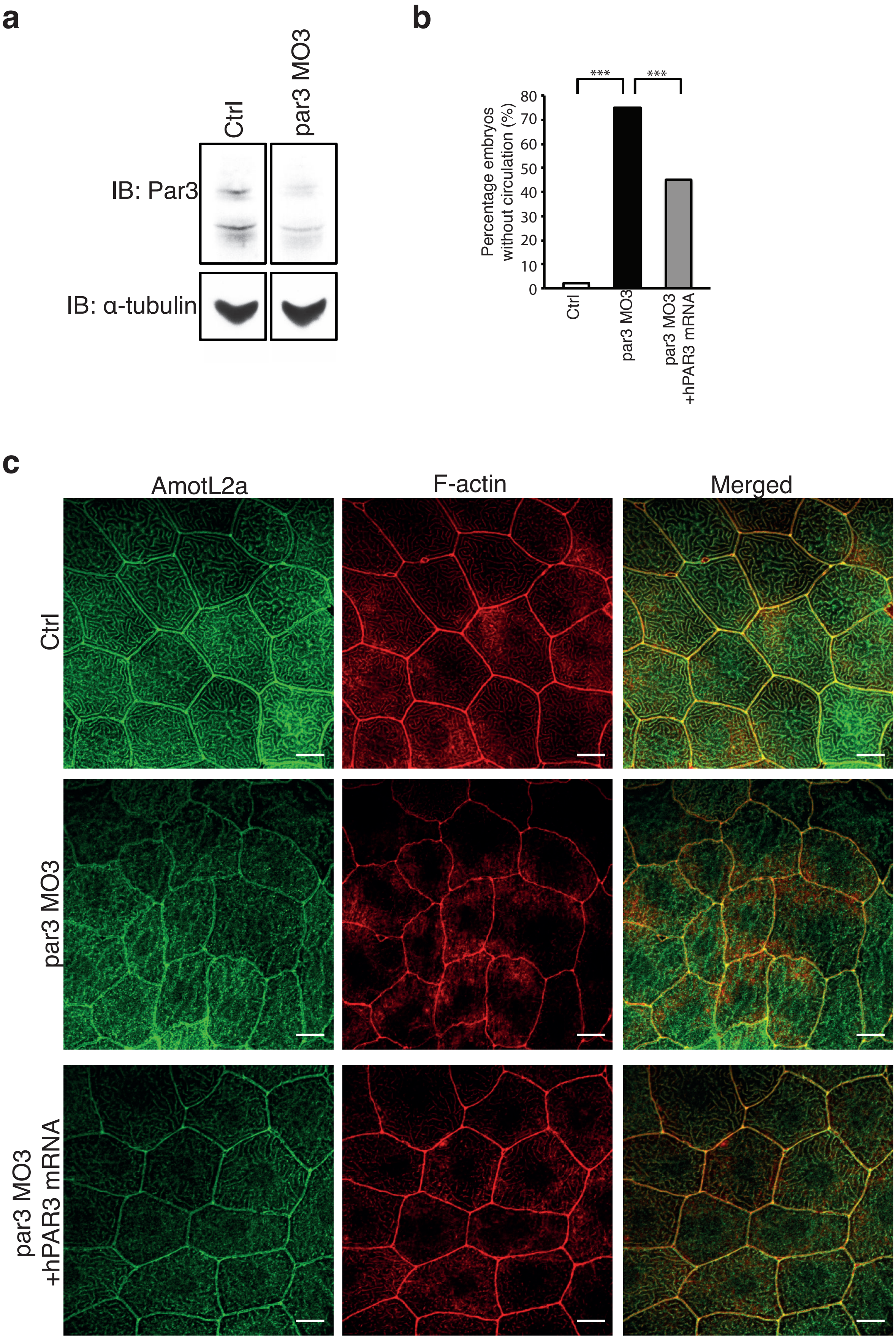

Hultin et al, Supplementary Figure 3

Full Blots to Figure 1d

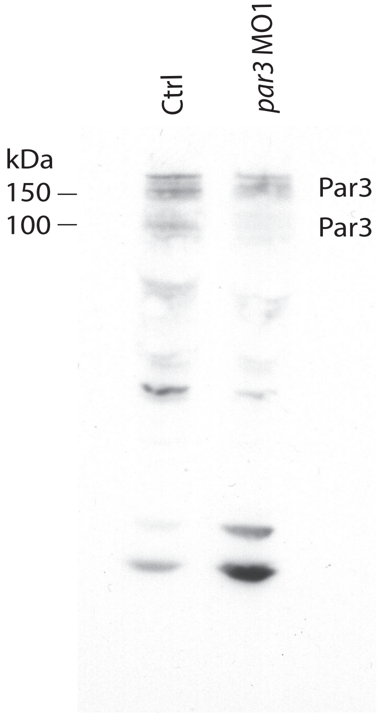

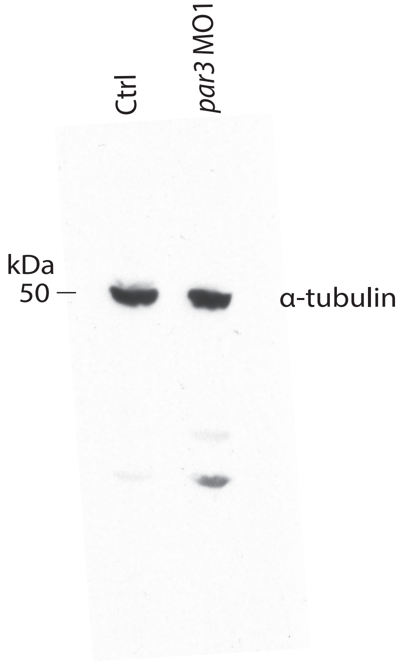

Hultin et al, Supplementary Figure 4

Full Blots to Figure 3 b & d

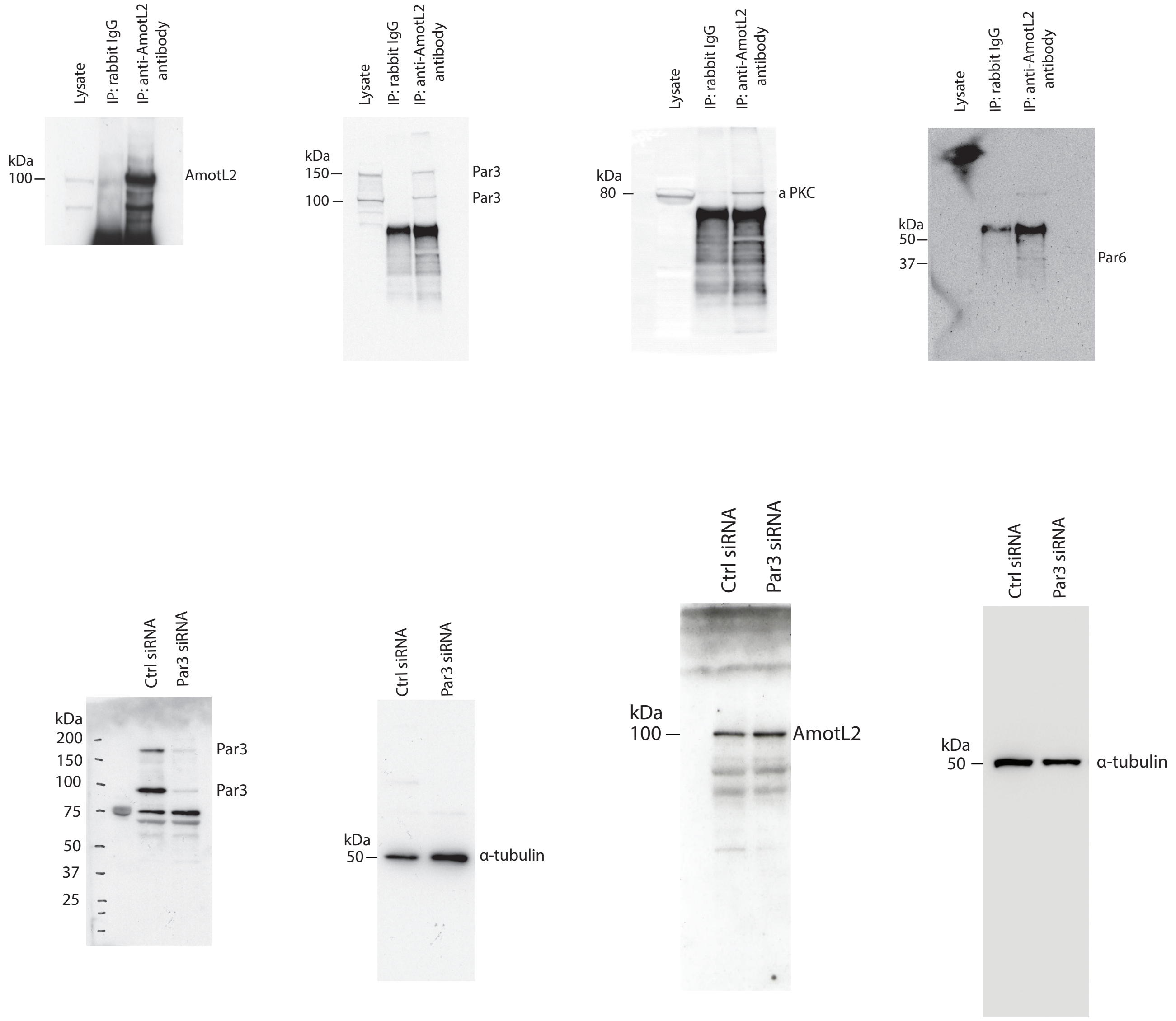

Hultin et al, Supplementary Figure 5

Full Blots to Figure. 4 e

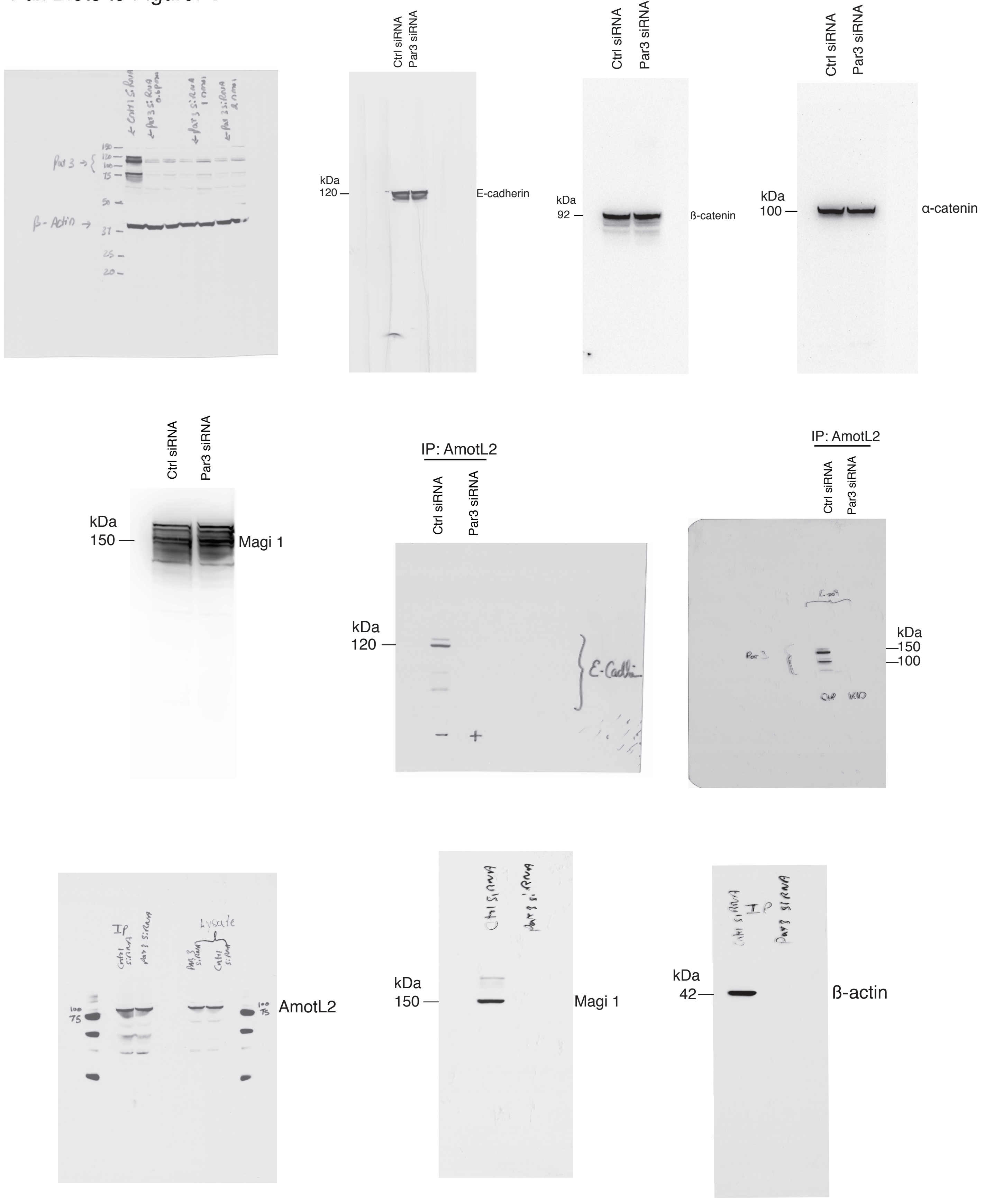

Hultin et al, Supplementary Figure 6

Full blots to Supplementary figure 1a

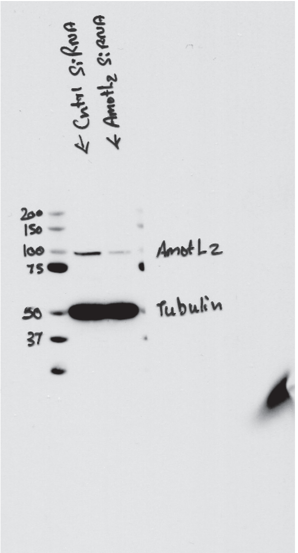

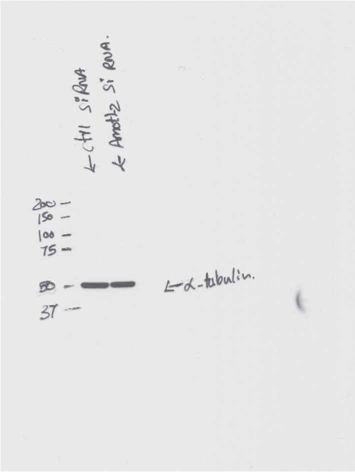

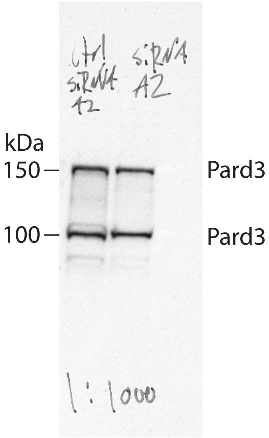

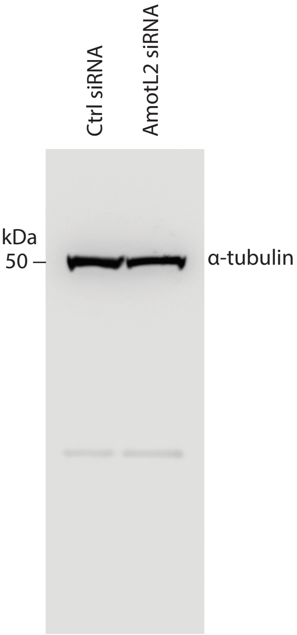

Hultin et al, Supplementary Figure 7

Full blots to Supplementary Figure 3 a

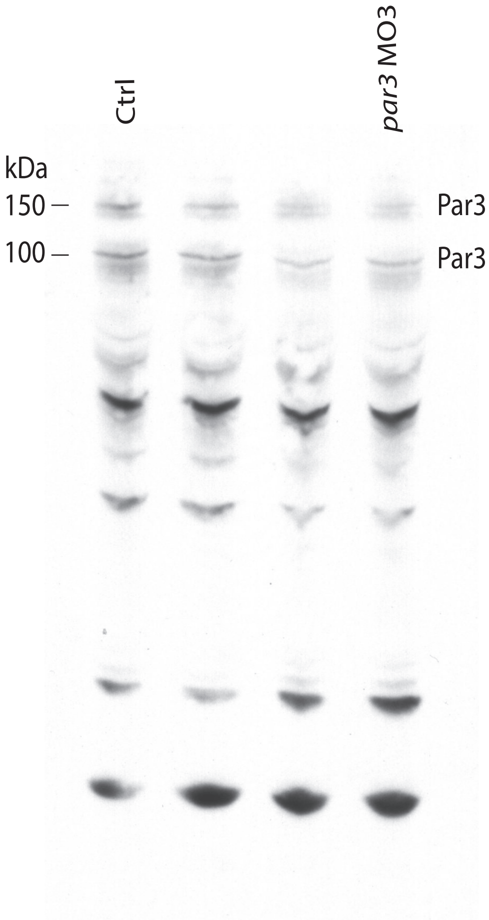

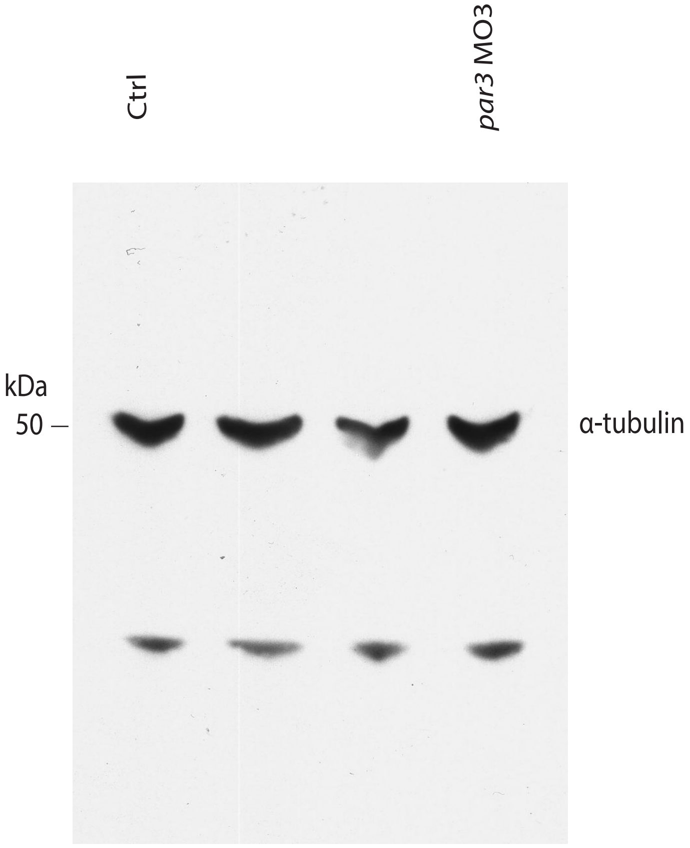

Hultin et al, Supplementary Figure 8
